# Doxycycline Changes the Transcriptome Profile of mIMCD3 Renal Epithelial Cells

**DOI:** 10.3389/fphys.2021.771691

**Published:** 2021-11-05

**Authors:** Hyun Jun Jung, Richard Coleman, Owen M. Woodward, Paul A. Welling

**Affiliations:** ^1^Division of Nephrology, Department of Medicine, Johns Hopkins University School of Medicine, Baltimore, MD, United States; ^2^Department of Physiology, University of Maryland School of Medicine, Baltimore, MD, United States; ^3^Department of Physiology, Johns Hopkins University School of Medicine, Baltimore, MD, United States

**Keywords:** doxycycline, RNA-seq, transcriptional response, cell proliferation, mIMCD3

## Abstract

Tetracycline-inducible gene expression systems have been used successfully to study gene function *in vivo* and *in vitro* renal epithelial models but the effects of the common inducing agent, doxycycline (DOX), on gene expression are not well appreciated. Here, we evaluated the DOX effects on the transcriptome of a widely used renal epithelial cell model, mIMCD3 cells, to establish a reference. Cells were grown on permeable filter supports in the absence and presence of DOX (3 or 6 days), and genome-wide transcriptome profiles were assessed using RNA-Seq. We found DOX significantly altered the transcriptome profile, changing the abundance of 1,549 transcripts at 3 days and 2,643 transcripts at 6 days. Within 3 days of treatment, DOX significantly decreased the expression of multiple signaling pathways (ERK, cAMP, and Notch) that are associated with cell proliferation and differentiation. Genes associated with cell cycle progression were subsequently downregulated in cells treated with DOX for 6 days, as were genes involved in cellular immune response processes and several cytokines and chemokines, correlating with a remarkable repression of genes encoding cell proliferation markers. The results provide new insight into responses of renal epithelial cells to DOX and a establish a resource for DOX-mediated gene expression systems.

## Introduction

Gene expression systems utilizing drug-induced trans-activation provide the means to conditionally investigate gene function in a temporal manner. They can be used to activate expression of a target gene for study, or to induce Cre-recombinase for conditional knockout cell of floxed alleles. The tetracycline-inducible gene expression system has proven especially popular. Doxycycline (DOX), a synthetic derivative of tetracycline, is widely used in these systems (Kistner et al., 1996; Das et al., 2016) because of its long half live and low cell toxicity. However, effects of DOX on a global gene expression and cellular processes in renal epithelial cell models are not known.

The absence of DOX effects on global gene expression in *Saccharomyces cerevisiae* (Wishart et al., 2005) initially suggested DOX might be inert. However, several more recent studies revealed DOX can change gene expression in mammalian tissues. For example, in *ex vivo* studies with surgically removed pterygia tissue from the eye, RNA sequencing (RNA-Seq)-based transcriptomic analysis (Larrayoz et al., 2012) revealed DOX affected the expression of mitochondrial genes, the ER stress cascade, growth factors, interleukins, cell cycle regulators, integrins, and components of the extracellular matrix. In rat aortic tissue (Lu et al., 2017), DOX was found to change the expression of other sets of genes, and these were mainly enriched in pathways that control neutrophil chemotaxis, chronic inflammatory responses, and cellular responses to mechanical stimuli and negatively regulate apoptotic processes. *In vivo* effects of DOX have been documented in studies to characterize the trans-activator (rtTA2) system in the mouse liver (Reboredo et al., 2008), and DOX-sensitive genes were found to enriched in cellular pathways related to cell growth and death and mitochondrial electron transport (Reboredo et al., 2008). Although the sample size still remains small, these few studies question the extent to which DOX may have generalizable responses; the heterogenous nature of responses suggest DOX action may be specifically governed by cell type or experimental condition. More studies are required with other cell types to identify common pathways. It remains unknown if DOX affects gene expression in renal epithelial cells.

Here we focus on characterizing the DOX response in the renal epithelial cell line, mIMCD3. The cells have been engineered to include a tetracycline-inducible gene expression system (Schlimpert et al., 2018; Lashhab et al., 2019; Kang et al., 2019), and the model has proven popular for studying genes in a renal epithelial environment. Here we examine how DOX affects the transcriptome profiles of mIMCD3 cells with RNA-Seq. The data should provide an informative resource for future studies with the tetracycline-inducible mIMCD3 cell line and similar *in vitro* renal epithelial models.

## Materials and Methods

### Cell Culture

Mouse kidney epithelial mIMCD3 cells derived from the inner medulla of a simian virus SV40 transgenic mouse were obtained from Maryland Polycystic Kidney Disease Research and Translation Core Center at the University of Maryland, and documented to be mycoplasma free. Cells were grown in T75 flasks with the RenaLife Epithelial Basal Medium (LM-0010, LIFELINE CELL TECHNOLOGY) supplemented with a RenaLife LifeFactors Kit (LS-1048, LIFELINE CELL TECHNOLOGY) and 5% FBS. This culture medium, which contains a nutrient blend of amino acids, vitamins, organic and inorganic supplements and salts, growth factors (0.5 μg/mL Insulin, 1 μM Epinephrine, 0.1 μg/mL Hydrocortisone, 10 nM Triiodothyronine, 10 ng/mL EGF, 5 μg/mL Transferrin, 2.4 mM L-Alanyl-L-Glutamine), and antibiotics (30 μg/mL Gentamicin, 15 ng/mL Amphotericin B), has become the choice for *in vitro* studies in the Maryland Cell engineering core of the NIH U54 funded Polycystic Kidney Disease Research Consortium. It was used here to provide a reference for on-going and future transcriptome profiling studies in the core. For RNA-Seq studies, cells were plated permeable filters (Polyester membrane) of a 6-well transwell plate (#3450, CORNING) and grown to confluence (10 days) before doxycycline treatment. Doxycycline (2 μg/mL) or vehicle (DMSO) added to both sides of the permeable filter and changed daily for 3 or 6 days.

### Total RNA Isolation and RNA Sequencing-Based Transcriptome Profiling

Cells were lysed in Trizol reagent (15596018, Invitrogen). Total RNA was isolated from the Trizol lysate using Direct-Zol RNA Miniprep plus kit (R2070, ZYMO RESEARCH) and eluted in RNase-free water, and RNA concentration was measured using Qubit^TM^ RNA HS Assay Kit (Q32852, Invitrogen). To enrich mRNA, 1–3 μg of total RNA was applied to oligo dT-based mRNA isolation using NEBNext^®^ Poly(A) mRNA Magnetic Isolation Module (E7490, NEW ENGLAND BioLabs Inc.) according to manufacturer’s instructions. mRNA (20 ng) was used to create the cDNA libraries, using NEBNext^®^ Ultra II Directional RNA Library Prep Kit for Illumina (E7760, NEW ENGLAND BioLabs Inc.) and NEBNext^®^ Multiplex Oligos (E7335; E7500, NEW ENGLAND BioLabs Inc.). cDNA libraries were sequenced on the Illumina HiSeq 4,000 platform. Sequence reads (2 × 75 bp, paired-end) were aligned on *Ensembl* genome GRCm38p6 using STAR (2.6.0c).

### Differential Expression Analysis and Bioinformatic Analysis

To identify differentially expressed genes between vehicle- and doxycycline-treated cells, transcript abundance was quantified using *salmon* (Patro et al., 2017). Differential expression (DE) analysis was carried out using *edgeR* (Robinson et al., 2010) on *R* (3.6.0). Low abundant genes with CPM (Counts Per Million) less than 1 were removed from the data set for the DE analysis. Significance of DE was determined using a modified statistical test (*edgeR* “glmTreat”) with a threshold of expression changes above 20% at FDR < 0.05 (Chen et al., 2016). Plots were generated using R package *ggplot2*. Pathway enrichment analysis of DE genes was carried out using *Gene Ontology* (Biological process) on *Metascape* platform^1^ (Zhou et al., 2019). The GO term analysis was performed using NaviGO (Wei et al., 2017).

### Statistics

Significance of DE was evaluated using *edgeR* and *p*-values were corrected using the Benjamini-Hochberg method. For significance of gene enrichment in the pathway analysis, *q* < 0.05 was considered as significant enrichment.

### Data Availability

All fastq files and a raw count file from RNA-Seq were deposited in GEO (GSE171573^2^).

## Results

### Transcriptome Profiles of mIMCD3 on Different Culture Environments

RNA-Seq analysis was performed to provide a transcript database of fully polarized mIMCD3 cells on permeable filter supports in the RenalLife medium which includes growth factors and antibiotics. For these studies, the cells were grown to confluence on the permeable filter supports for 10 days, and then were treated with DMSO or DOX in the RenalLife medium for 3 or 6 days.

**FIGURE 1:**
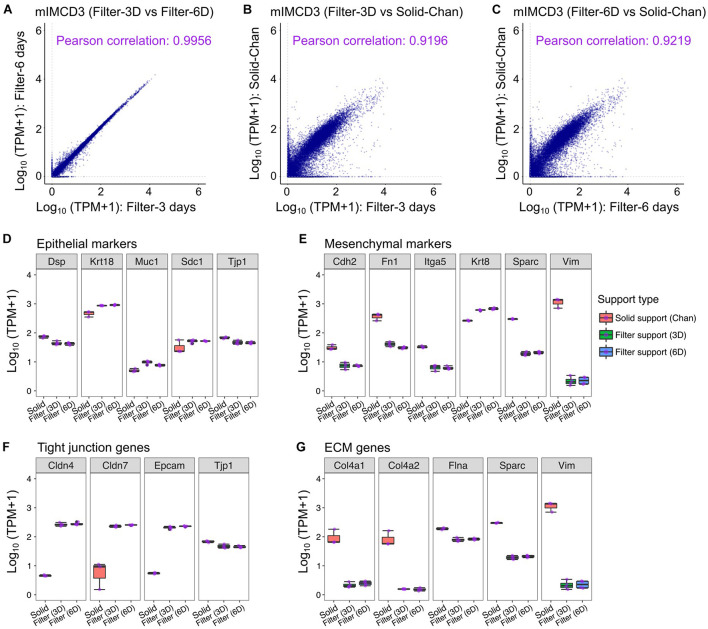
Transcriptomic characteristics of renal epithelial cell line mIMCD3 in different culture conditions. **(A)** Transcriptomic correlation was assessed in mIMCD3 cells grown on the permeable filter supports for 3 and 6 days. **(B,C)** Transcriptome profiles of mIMCD3 grown on the permeable filter support and the solid support were compared. The transcriptome dataset of mIMCD3 cells grown on the solid support was obtained from GSE97770 (Chan et al., 2018). **(D–G)** Box plots of gene expression associated with epithelial cell, mesenchymal cell, tight junction formation, and extracellular matrix (ECM) in mIMCD3 cells grown on the solid support (Chan et al., 2018) and the permeable filter support.

To establish the baseline, we first examined the control cells, treated with DMSO. We found a high correlation between the transcriptome of cells treated with DMSO at 3 and 6 days (Pearson correlation: 0.9956, Figure 1A), consistent with stable gene expression and lack of transient DMSO responses. Based comparison of normalized expression values (transcripts per million, TPM), the transcriptome exhibited a high correlation with native mIMCD3 cells grown on plastic solid supports (GSE97770) as reported by Chan et al. (2018) (Figures 1B,C). Whether grown on permeable supports or plastic solid supports, mIMCD3 equally express the conventional epithelial cell markers (Jedroszka et al., 2017), such as *Tjp1*, *Krt18*, *Dsp*, *Muc1*, and *Sdc1* (Figure 1D). Other extracellular matrix components, *Col4a2* and *Sparc*, were also more abundantly expressed in the mIMCD3 cells grown on the solid support compared to mIMCD3 cells on the filter support (Figure 1G). Interestingly, mesenchymal cell marker genes, namely *Cdh2*, *Fn1*, *Sparc*, and *Vim*, were also more abundant in mIMCD3 cells grown on the solid support compared to mIMCD3 cells grown on the filter support (Figure 1E). By contrast, in mIMCD3 cells grown on the filter supports, tight junction proteins, *Cldn4*, *Cldn7*, and *Epcam*, were more abundant than in cells on the solid support (Figure 1F). Thus, transcriptomic signature of mIMCD3 cells is most compatible with a differentiated epithelial state when cells are grown on permeable supports compared to cells grown on plastic supports. Yet the transcriptome profiles of mIMCD3 cells grown on permeable and non-permeable are more similar than different. Interestingly, we did not identify the enrichment of specific transcripts of medullary collecting duct in this cell line.

Nevertheless, the dataset of comprehensive transcriptome profile in mIMCD3 provides an informative resource for further utilization as the *in vitro* kidney epithelial cell model. To expand understanding of intracellular signaling pathways in mIMCD3 cells grown on solid supports (Valkova and Kultz, 2006; Chan et al., 2018) compared to cells grown on filters, we classified transcriptome profile into genes encoding transcription factors, ion channels, transporters, and G protein-coupled receptors (GPCRs) in Tables 1–4 (Supplementary Table 1). Many of the listed genes have been identified in previous studies. For example, *Hnf1b* (hepatocyte nuclear factor-1 beta, TPM: 43.0) (Aboudehen et al., 2017), *Pax2* (Paired box 2, TPM: 20.8) (Torban et al., 2000; Cai et al., 2005), and *Egr1* (Early Growth Response 1, TPM: 49.7) (Cohen et al., 1994, 1996) are transcription factors reliably expressed in the mIMCD3 cells (Supplementary Table 1, “Transcription factors”). In addition to known expression of transcription factors, expression of transcription factors that could be involved in the kidney nephron development, such as *Emx2*, *Pax8*, *Tfap2a*, *Hmga2*, *Hmgb2*, and *Hoxa11*, were identified (Ribes et al., 2003; Schwab et al., 2003; Chambers et al., 2019).

**TABLE 1:** Top 30 transcription factors (see the full transcription factor list in Supplementary Table 1).

**Gene symbol**	**Gene name**	**TF family**	**Mean (TPM)**	**S.E.M.**	**Rank**
*Ybx1*	Y box protein 1	CSD	696.7	8.1	211
*Atf4*	Activating transcription factor 4	TF_bZIP	489.0	18.8	269
*Id2*	Inhibitor of DNA binding 2	bHLH	374.5	17.0	328
*Hmgb1*	High mobility group box 1	HMG	339.6	19.4	354
*Hmga1*	High mobility group AT-hook 1	HMGI/HMGY	331.4	3.9	362
*Ddit3*	DNA-damage inducible transcript 3	TF_bZIP	326.8	7.5	365
*Glmp*	Glycosylated lysosomal membrane protein	NCU-G1	296.5	13.1	397
*Csde1*	Cold shock domain containing E1, RNA binding	CSD	291.0	8.5	406
*Sub1*	SUB1 homolog (*S*. *cerevisiae*)	PC4	261.5	8.5	458
*Ybx3*	Y box protein 3	CSD	252.0	2.6	477
*Tsc22d1*	TSC22 domain family, member 1	TSC22	202.8	6.2	592
*Ssrp1*	Structure specific recognition protein 1	HMG	191.3	5.6	628
*Jund*	Jun D proto-oncogene	TF_bZIP	190.8	6.7	631
*Zbtb18*	Zinc finger and BTB domain containing 18	ZBTB	181.0	12.0	666
*Cers2*	Ceramide synthase 2	Homeobox	169.6	6.8	718
*Hmgb2*	High mobility group box 2	HMG	159.0	6.4	763
*Litaf*	LPS-induced TN factor	zf-LITAF-like	147.0	4.9	822
*Gatad1*	GATA zinc finger domain containing 1	zf-GATA	139.2	2.3	877
*Smarce1*	SWI/SNF related, matrix associated, actin dependent regulator of chromatin, subfamily e, member 1	HMG	136.5	5.3	898
*Junb*	Jun B proto-oncogene	TF_bZIP	130.5	6.1	950
*Cux1*	Cut-like homeobox 1	CUT	126.4	2.8	979
*Bhlhe40*	Basic helix-loop-helix family, member e40	bHLH	124.3	5.4	1,000
*Tfdp1*	Transcription factor Dp 1	E2F	109.1	3.2	1,131
*Pax8*	Paired box 8	PAX	107.5	3.0	1,152
*Gtf3a*	General transcription factor III A	zf-C2H2	103.7	1.7	1,198
*Zfp91*	Zinc finger protein 91	zf-C2H2	101.8	2.9	1,222
*Hmg20b*	High mobility group 20B	HMG	97.2	3.4	1,284
*Mbd3*	Methyl-CpG binding domain protein 3	MBD	96.2	3.5	1,297
*Trp53*	Transformation related protein 53	P53	96.0	3.1	1,303
*Irf7*	Interferon regulatory factor 7	IRF	91.1	2.5	1,379
*Atf5*	Activating transcription factor 5	TF_bZIP	83.0	3.8	1,543
*Mbd2*	Methyl-CpG binding domain protein 2	MBD	81.4	3.3	1,573
*Ncor1*	Nuclear receptor co-repressor 1	MYB	80.9	4.2	1,582

**TABLE 2:** Top 30 ion channels (see the full ion channel list in Supplementary Table 1).

**Gene symbol**	**Gene name**	**Class**	**Mean (TPM)**	**S.E.M.**	**Rank**
*Vdac1*	Voltage-dependent anion channel 1	Voltage dependent anion channels	269.45	4.56	441
*Clic1*	Chloride intracellular channel 1	Chloride intracellular channels	232.35	4.91	517
*Vdac3*	Voltage-dependent anion channel 3	Voltage dependent anion channels	177.17	5.61	687
*Vdac2*	Voltage-dependent anion channel 2	Voltage dependent anion channels	160.05	4.23	757
*Kcnk1*	Potassium channel, subfamily K, member 1	Potassium two pore domain channel subfamily K	90.09	3.46	1,393
*Cacnb3*	Calcium channel, voltage-dependent, beta 3 subunit	Calcium voltage-gated channel auxiliary beta subunits	59.26	2.19	2,174
*Mcoln1*	Mucolipin 1	Transient receptor potential cation channels	50.48	2.50	2,573
*Clic4*	Chloride intracellular channel 4 (mitochondrial)	Chloride intracellular channels	40.51	1.32	3,207
*Clcn3*	Chloride channel, voltage-sensitive 3	Chloride voltage-gated channels	40.19	0.95	3,227
*Trpv4*	Transient receptor potential cation channel, subfamily V, member 4	Transient receptor potential cation channels	29.76	1.38	4,145
*Lrrc8d*	Leucine rich repeat containing 8D	Volume regulated anion channel subunits	29.74	1.06	4,149
*Ano1*	Anoctamin 1, calcium activated chloride channel	Anoctamins	23.78	0.57	4,972
*Piezo1*	Piezo-type mechanosensitive ion channel component 1	Mechanosensitive Piezo Ion Channels	23.69	1.31	4,993
*Trpm7*	Transient receptor potential cation channel, subfamily M, member 7	Transient receptor potential cation channels	23.67	1.20	4,995
*Ano6*	Anoctamin 6	Anoctamins	20.91	1.05	5,396
*Pkd2*	Polycystic kidney disease 2	Transient receptor potential cation channels	20.65	0.77	5,441
*Clcn7*	Chloride channel, voltage-sensitive 7	Chloride voltage-gated channels	18.62	0.92	5,830
*Tpcn1*	Two pore channel 1	Two pore segment channels	16.83	0.63	6,237
*Kcnk5*	Potassium channel, subfamily K, member 5	Potassium two pore domain channel subfamily K	16.82	0.63	6,243
*Lrrc8b*	Leucine rich repeat containing 8 family, member B	Volume regulated anion channel subunits	15.28	0.64	6,604
*Trpm4*	Transient receptor potential cation channel, subfamily M, member 4	Transient receptor potential cation channels	13.87	0.60	6,909
*Ano10*	Anoctamin 10	Anoctamins	13.73	0.47	6,952
*Itpr3*	Inositol 1,4,5-triphosphate receptor 3	Inositol 1,4,5-triphosphate receptors	12.86	0.47	7,187
*Ano9*	Anoctamin 9	Anoctamins	12.67	0.91	7,244
*Kcnj16*	Potassium inwardly rectifying channel, subfamily J, member 16	Potassium inwardly rectifying channel subfamily J	12.52	0.83	7,289
*Clcn4*	Chloride channel, voltage-sensitive 4	Chloride voltage-gated channels	9.46	0.43	8,239
*Lrrc8a*	Leucine rich repeat containing 8A	Volume regulated anion channel subunits	9.42	0.43	8,249
*Itpr1*	Inositol 1,4,5-trisphosphate receptor 1	Inositol 1,4,5-triphosphate receptors	8.12	0.48	8,722
*Clcn5*	Chloride channel, voltage-sensitive 5	Chloride voltage-gated channels	7.65	0.28	8,888
*Ano8*	Anoctamin 8	Anoctamins	6.10	0.27	9,514

**TABLE 3:** Top 30 transporters (see the full transporter list in Supplementary Table 1).

**Gene symbol**	**Gene name**	**Class**	**Mean (TPM)**	**S.E.M.**	**Rank**
*Slc25a3*	Solute carrier family 25 (mitochondrial carrier, phosphate carrier), member 3	Solute carriers	542.86	2.91	248
*Atp1a1*	ATPase, Na^+^/K^+^ transporting, alpha 1 polypeptide	ATPase Na^+^/K^+^ transporting subunits	314.46	7.63	376
*Slc3a2*	Solute carrier family 3 (activators of dibasic and neutral amino acid transport), member 2	Solute carriers	257.29	11.12	465
*Slc25a5*	Solute carrier family 25 (mitochondrial carrier, adenine nucleotide translocator), member 5	Solute carriers	239.92	4.94	498
*Atp1b1*	ATPase, Na^+^/K^+^ transporting, beta 1 polypeptide	ATPase Na^+^/K^+^ transporting subunits	197.25	7.02	610
*Slc25a4*	Solute carrier family 25 (mitochondrial carrier, adenine nucleotide translocator), member 4	Solute carriers	188.52	4.34	644
*Slc2a1*	Solute carrier family 2 (facilitated glucose transporter), member 1	Solute carriers	186.90	7.97	650
*Mtch2*	Mitochondrial carrier 2	Solute carriers	155.59	6.19	782
*Slc25a39*	Solute carrier family 25, member 39	Solute carriers	125.90	2.54	986
*Spns2*	Spinster homolog 2	Solute carriers	125.84	3.80	988
*Atp2a2*	ATPase, Ca^++^ transporting, cardiac muscle, slow twitch 2	ATPases Ca^2+^ transporting	101.64	3.64	1,224
*Ucp2*	Uncoupling protein 2 (mitochondrial, proton carrier)	Solute carriers	97.34	2.43	1,280
*Mpc2*	Mitochondrial pyruvate carrier 2	Solute carriers	96.23	4.44	1,296
*Slc30a9*	Solute carrier family 30 (zinc transporter), member 9	Solute carriers	92.35	2.38	1,357
*Mtch1*	Mitochondrial carrier 1	Solute carriers	86.90	1.63	1,455
*Tusc3*	Tumor suppressor candidate 3	Solute carriers	84.86	2.48	1,502
*Slc25a1*	Solute carrier family 25 (mitochondrial carrier, citrate transporter), member 1	Solute carriers	83.64	1.26	1,532
*Slc50a1*	Solute carrier family 50 (sugar transporter), member 1	Solute carriers	76.95	3.15	1,670
*Abcf2*	ATP-binding cassette, sub-family F (GCN20), member 2	ATP binding cassette subfamily F	70.26	2.23	1,822
*Slc38a2*	Solute carrier family 38, member 2	Solute carriers	69.63	2.95	1,844
*Slc39a1*	Solute carrier family 39 (zinc transporter), member 1	Solute carriers	67.52	1.21	1,896
*Abce1*	ATP-binding cassette, sub-family E (OABP), member 1	ATP binding cassette subfamily E	67.42	2.99	1,899
*Slc25a24*	Solute carrier family 25 (mitochondrial carrier, phosphate carrier), member 24	Solute carriers	66.16	2.28	1,938
*Slc35b1*	Solute carrier family 35, member B1	Solute carriers	64.59	1.89	1,987
*Slc39a7*	Solute carrier family 39 (zinc transporter), member 7	Solute carriers	61.09	2.31	2,108
*Slc25a11*	Solute carrier family 25 (mitochondrial carrier oxoglutarate carrier), member 11	Solute carriers	57.79	2.34	2,234
*Slc25a17*	Solute carrier family 25 (mitochondrial carrier, peroxisomal membrane protein), member 17	Solute carriers	56.16	1.54	2,284
*Slc35a4*	Solute carrier family 35, member A4	Solute carriers	55.69	3.65	2,306
*Abcf1*	ATP-binding cassette, sub-family F (GCN20), member 1	ATP binding cassette subfamily F	53.81	1.98	2,386
*Slc44a2*	Solute carrier family 44, member 2	Solute carriers	52.71	1.49	2,458

**TABLE 4:** Top 30 GPCRs (see the full GPCR list in Supplementary Table 1).

**Gene symbol**	**Gene name**	**Class**	**Mean (TPM)**	**S.E.M.**	**Rank**
*Adgrg1*	Adhesion G protein-coupled receptor G1	Adhesion G protein-coupled receptors, subfamily G	93.06	6.39	1,343
*Gprc5c*	G protein-coupled receptor, family C, group 5, member C	G protein-coupled receptors, Class C orphans	87.39	2.36	1,443
*Gprc5a*	G protein-coupled receptor, family C, group 5, member A	G protein-coupled receptors, Class C orphans	74.50	2.49	1,719
*F2r*	Coagulation factor II (thrombin) receptor	F2R receptors	43.62	1.65	2,992
*Tpra1*	Transmembrane protein, adipocyte associated 1	7TM orphan receptors	34.63	0.79	3,641
*Adgrl2*	Adhesion G protein-coupled receptor L2	Adhesion G protein-coupled receptors, subfamily L	29.26	1.34	4,220
*F2rl1*	Coagulation factor II (thrombin) receptor-like 1	F2R receptors	23.20	1.07	5,055
*Adgre5*	Adhesion G protein-coupled receptor E5	Adhesion G protein-coupled receptors, subfamily E	23.00	0.83	5,088
*Smo*	Smoothened, frizzled class receptor	G protein-coupled receptors, Class F frizzled	22.86	0.74	5,106
*Gpr137*	G protein-coupled receptor 137	7TM orphan receptors	21.52	0.75	5,291
*Gpr108*	G protein-coupled receptor 108	7TM orphan receptors	21.06	0.88	5,368
*Fzd6*	Frizzled class receptor 6	G protein-coupled receptors, Class F frizzled	20.98	0.82	5,385
*Gpr107*	G protein-coupled receptor 107	7TM orphan receptors	19.89	1.06	5,600
*Gabbr1*	Gamma-aminobutyric acid (GABA) B receptor, 1	Gamma-aminobutyric acid type B receptor subunits	16.97	1.36	6,202
*Adora1*	Adenosine A1 receptor	Adenosine receptors	15.32	0.64	6,594
*Lgr4*	Leucine-rich repeat-containing G protein-coupled receptor 4	G protein-coupled receptors, Class A orphans	13.87	0.54	6,910
*Adgra3*	Adhesion G protein-coupled receptor A3	Adhesion G protein-coupled receptors, subfamily A	12.81	0.97	7,206
*Ptger4*	Prostaglandin E receptor 4 (subtype EP4)	Prostaglandin receptors	11.67	0.43	7,534
*Gpr161*	G protein-coupled receptor 161	G protein-coupled receptors, Class A orphans	11.52	0.34	7,593
*Gpr19*	G protein-coupled receptor 19	G protein-coupled receptors, Class A orphans	11.31	0.50	7,664
*Celsr2*	Cadherin, EGF LAG seven-pass G-type receptor 2	Adhesion G protein-coupled receptors, subfamily C	11.29	0.89	7,667
*Adgrg6*	Adhesion G protein-coupled receptor G6	Adhesion G protein-coupled receptors, subfamily G	10.66	0.69	7,856
*Gpr160*	G protein-coupled receptor 160	G protein-coupled receptors, Class A orphans	10.30	0.50	7,948
*Fzd1*	Frizzled class receptor 1	G protein-coupled receptors, Class F frizzled	9.19	0.31	8,316
*Fzd7*	Frizzled class receptor 7	G protein-coupled receptors, Class F frizzled	7.65	0.27	8,887
*P2ry2*	Purinergic receptor P2Y, G-protein coupled 2	P2Y receptors	6.67	0.17	9,291
*Gpr39*	G protein-coupled receptor 39	G protein-coupled receptors, Class A orphans	5.72	0.20	9,673
*Pitpnm3*	PITPNM family member 3	Atypical chemokine receptors	5.70	0.40	9,679
*Lgr6*	Leucine-rich repeat-containing G protein-coupled receptor 6	G protein-coupled receptors, Class A orphans	5.35	0.33	9,813
*Gpr146*	G protein-coupled receptor 146	G protein-coupled receptors, Class A orphans	5.16	0.25	9,903

As shown in the previous studies (Slaats et al., 2015; Liu et al., 2018), expression of two ion transport proteins, *Pkd2* (Polycystic kidney disease 2, TPM: 20.6) and *Clcn4* (H^+^/Cl^–^ exchange transporter 4, TPM: 9.5), were found in the current transcriptome (Table 2). We also identified the mechanosensitive cation channel *Piezo1* (piezo-type mechanosensitive ion channel component 1) (Coste et al., 2010; Table 2). Expression of ion channels, *Trpm4* (Transient Receptor Potential Cation Channel Subfamily M Member 4, TPM: 13.9), *Trpm6* (Transient Receptor Potential Cation Channel Subfamily M Member 6, TPM: 3.3), and *Trpm7* (Transient Receptor Potential Cation Channel Subfamily M Member 7, TPM: 23.7), as well as a Mg^+^ transporter, *Magt1* (TPM: 20.1), suggests further utilization as an *in vitro* model for studying transepithelial Mg^+^ transport mechanisms in kidney epithelial cells (Groenestege et al., 2006; Tables 2, 3 and Supplementary Table 1, “Ion channels” and “Transporters”).

The mIMCD3 cell line has been widely used in several studies of primary cilia conformation and function. The current transcriptome profile provides information about ciliary GPCRs expressed in mIMCD3 cells. Among the ciliary GPCRs, as previously reported (Hilgendorf et al., 2016; Mykytyn and Askwith, 2017), we identified expression of *Gpr161* (G protein-coupled receptor 161, TPM: 11.5), *Ptger4* (prostaglandin E receptor 4, TPM: 11.7), and *Smo* (Smoothened homolog, TPM: 22.86) in mIMCD3 cells (Table 4). In addition to ciliary GPCRs, expression of two adenylyl cyclases, *Adcy1* (TPM: 8.9) and *Adcy6* (TPM: 31.5), was found in the transcriptome profile. These adenylyl cyclases could be considered in further studies for examining cAMP responses in mIMCD3 cells (Strait et al., 2010).

### Doxycycline-Responsive Transcriptome Changes in mIMCD3 Cells

**FIGURE 2:**
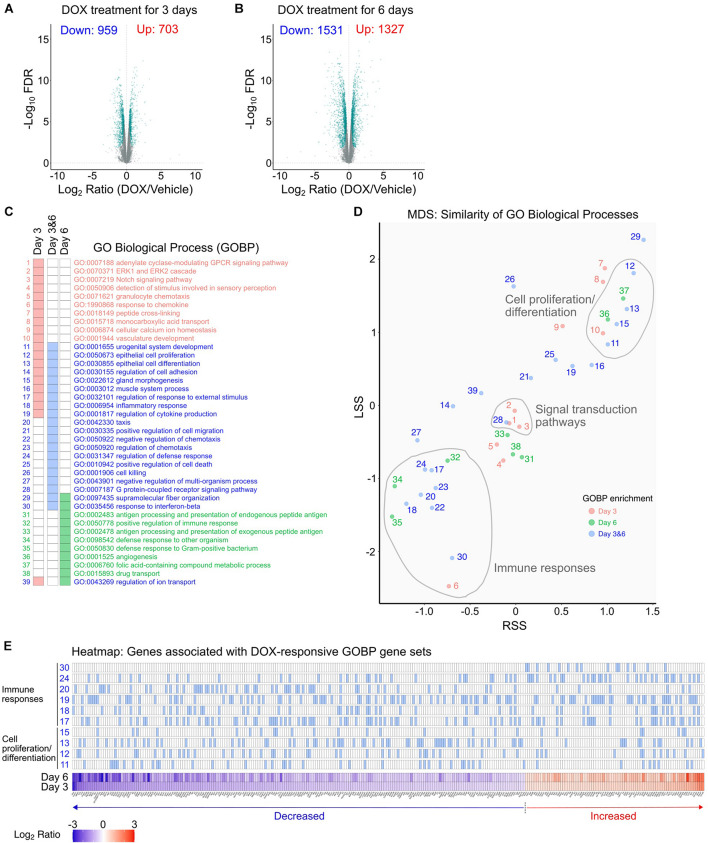
DOX-responsive transcriptomic changes in mIMCD3 cells. **(A,B)** Identification of transcriptomic changes in mIMCD3 cells treated with DOX for 3 and 6 days using RNA-Seq-based differential expression analysis. Significantly changed genes (FDR < 0.05 over 20% expression change threshold) were labeled as green in volcano plots and used further downstream analysis. **(C)** Gene Ontology (GO) biological processes significantly enriched by DOX-responsive genes. Cellular processes (GO Biological Process) were identified from significantly changed genes by DOX treatment for 3 and 6 days. To avoid sample-source bias, whole transcriptome was used as a background gene set. Significant enrichment was considered as *q* < 0.05. **(D)** Bubble chart view of the GO term association using *NaviGO*. In the plot, the *X*-axis is the Resnik semantic similarity score (RSS) and the *Y*-axis chosen is Lin’s similarity score (LSS). GO terms are colored according to time point when DE genes are found, day 3, pink; day 6: green; and day 3 and 6: blue. The number of GO terms are listed in **(C)**. **(E)** Heatmap of gene sets associated with “cell proliferation/differentiation” and “immune responses.” Full list of genes associated with GO terms are provided in Supplementary Table 4.

To characterize the DOX response in mIMCD3 cells, we compared the transcriptomes of DOX-treated mIMCD3 cells and DMSO-treated mIMCD3 cells (Figure 2 and Supplementary Table 2). We found DOX changed the gene expression profile, reflecting a change in abundance of 1,662 genes at 3 days (Figure 2A) and 2,858 genes at 6 days of treatment (Figure 2B). A total of 1,157 genes were consistently changed at both times. Downstream analysis identified GO Biological Processes (GOBPs) that are enriched in DOX-treated cells (Figure 2C). The *NaviGO* based GO term association analysis, which classifies similarity within GO term hierarchies, revealed three major clusters of GOBPs: (1) cell proliferation/differentiation; (2) signal transduction; and (3) immune responses (Figure 2D). In the cells treated with DOX for 3 days, differentially expressed genes were enriched for signal transduction pathways and cell proliferation/differentiation (Figures 2C,D). In particular, this involved significant changes of genes associated with ERK, cAMP, and Notch signaling pathways, known to mainly involve cell proliferation and development processes (Figure 2C). Differentially expressed genes from the 6 days-dataset were also especially enriched in biological processes associated with cell proliferation/differentiation but were also enriched in immune response pathways (Figures 2C,D). As shown in the heatmap (Figure 2E), expression of genes involved in cell proliferation/differentiation processes and immune responses were mostly decreased at both times. The results indicate that DOX attenuates gene expression associated with cell proliferation and immune responses. Full list of genes associated with these pathways are provided in Supplementary Table 3.

**FIGURE 3:**
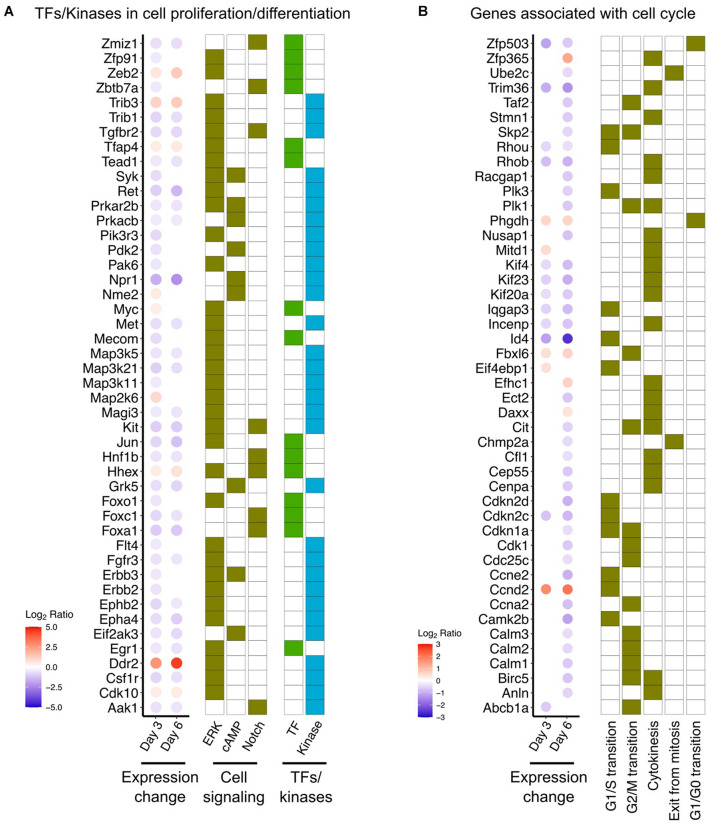
DOX-responsive genes associated with cell proliferation and differentiation. **(A)** DOX-responsive expressional change of transcription factors (TFs) and protein kinases in three signaling pathways (ERK, cAMP, and Notch). Full list of DOX-responsive genes involved in these signaling pathways was provided in Supplementary Table 3. **(B)** Expressional change of gene associated with cell cycle progression. Gene sets of each stage of cell cycle progression were obtained from Gene Ontology database (GO:0022402: cell cycle process).

Examination of genes encoding downstream factors in signal transduction processes, including transcription factors and kinases, revealed alterations in three different signaling pathways (cAMP, ERK, Notch). Remarkable downregulation of several transcription factors and protein kinases was found in DOX-treated cells (Figure 3A). Especially, *Hnf1b* (Chung et al., 2017), *Egr1* (Sukhatme et al., 1988), *Tead1* (Zhao et al., 2008), and members of the Fox family (Kume et al., 2000; Aschauer et al., 2013), which have been proposed previously as key players in epithelial cell proliferation. Additionally, several protein kinases downregulated in response to DOX, namely *Tgfbr2* (LeBleu et al., 2013), *Kit* (Gomes et al., 2018), and protein kinase A (Amsler et al., 1991), are highly related to regulation of epithelial cell function (Figure 3A). Furthermore, the effects of DOX to decrease the expression of genes involved in each step of the cell cycle and cell cycle progression suggest that DOX may have proclivity to inhibit cell proliferation (Figure 3B).

**FIGURE 4:**
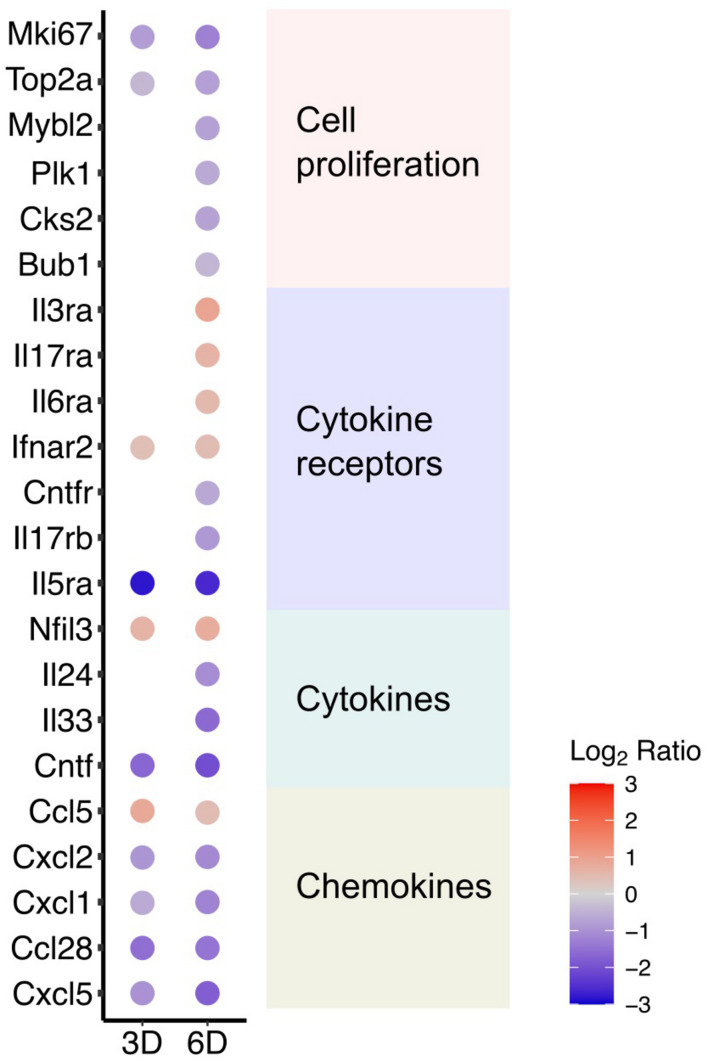
DOX-responsive cytokines and cell proliferation markers. Significantly changed genes encoding cytokines including chemokines were identified and listed in the plot. In addition, expressional changes of genes previously known as cell proliferation markers were listed in the plot.

The transcriptome dataset showing the attenuated cell cycle progression also exhibited that several genes known as cell proliferation markers were consistently downregulated by DOX treatment at 6 days (Figure 4). The result confirmed that DOX treatment suppresses cell proliferation. In addition to cell proliferation markers, DOX treatment for 3 and 6 days induced significant changes of immune response-associated cellular pathways (Figure 2), largely reflecting DOX-responsive reduction in cytokine production at day 3 and comprehensive alteration of cellular inflammatory response at day 6. In particularly, we identified several chemokines that were significantly downregulated at 3 days after DOX treatment from the literature-based gene sets of immunologic mediators (Commins et al., 2010; Figure 4), corresponding the pathways involved in the repression of epithelial cell proliferation, which are associated with epithelial responses to cytokines (Stadnyk, 1994).

## Discussion

The mIMCD3 cell line, an inner medullary collecting duct (IMCD) cell line derived from a simian virus 40 (SV40) T-antigen transgenic mice, has been widely used as an *in vitro* model to study renal epithelial physiology and cell biology (Rauchman et al., 1993; Yu et al., 2013; Flannery et al., 2015; Siroky et al., 2017). With inclusion of a DOX-inducible gene expression system, the cell line has become an invaluable model to study gene function in renal epithelial background. Here, we applied next-generation sequencing technology to characterize how the mIMCD3 transcriptome is affected by DOX with the goal of creating a database to guide interpretation of these studies.

Appropriate differentiation of epithelial cells and development of apical-basolateral polarity *in vitro* is commonly believed to require growth on a permeable surface. Comparison of the mIMCD3 transcriptome of cells grown on permeable polyester (PE) filter membrane surface, as defined here, with the published the transcriptome profile of mIMCD3 cells grown on plastic (Chan et al., 2018) lends credence to this idea. Although we found transcriptome profiles of mIMCD3 cells grown on filters exhibit a surprising degree of similarity with the transcriptome profile of cells grown on a solid surface (Reboredo et al., 2008), the profile of cells grown on filters is consistent with a more differentiated state. We found genes encoding key junctional stability components (Cldn4, Cldn7, Epcam) are induced when cells are grown on filters, whereas mesenchymal markers and ECM components were less abundant in filter-grown cells. Cldn4 (Claudin-4) and Cldn7 (Claudin-7) are among claudin family proteins that are specifically expressed in the loop of Henle (LOH) and distal nephron (Leiz and Schmidt-Ott, 2019), dictating the unique pericellular solute permeation profile and the electrical resistance of these nephron segments. Epcam (Epithelial Cell Adhesion Molecule) is best known as a marker of epithelial oncogenesis, but it is highly expressed in the LOH and collecting duct, where its natural role in cell adhesion and morphogenesis may determine the differentiated state of these nephron segments by interaction with Claudin-7 to negatively regulate epithelial migration by inhibiting ERK and actomyosin contractility (Barth et al., 2018). The increased abundances of ECM components may identify a gene expression signature of solid support grown cells, reflecting a futile cellular response to establish a permeable basement membrane, and thereby influence cell proliferation and differentiation (Matter et al., 2005; Ichikawa-Tomikawa et al., 2011; Bonnans et al., 2014; Diaz-Coranguez et al., 2019). Supporting this idea, the upregulated ECM are ubiquitous components of most basement membranes, including members of type IV collagen family proteins, Col4a1 and Col4a2, and the Secreted Protein Acidic and Rich in Cysteine (SPARC, aka Osteonectin).

We found the subtle growth substrate-dependent changes occur against an otherwise, remarkably stable mIMCD3 transcriptome profile. Constancy of the gene expression profile may explain why the phenotypic properties of the cell line have been so stable over time and numerous different studies. Comparison of the mIMCD3 transcriptome to the single cell RNA profiles of mouse cell kidney (Ransick et al., 2019) indicates that stability has come at the cost of lost nephron cell definition, however. The transcriptome profile of mIMCD3 does not uniquely resemble transcriptomes of any cell type in the kidney. Instead, it is compatible with a generic renal epithelial cell model, presumably reflecting a dedifferentiated state.

Tetracycline drugs, like Doxycycline (DOX), are widely used in eukaryotic cell gene expression systems, in part, because they have been assumed to have limited toxicity profiles. They inhibit protein synthesis in a broad spectrum of bacteria by binding to rRNAs of ribosomal complexes and double-strand RNAs (Chukwudi, 2016; Chukwudi and Good, 2016), but do not usually accumulate enough in mammalian cells to affect protein synthesis. We found DOX, at least at the higher concentrations commonly used *in vitro* cell models to drive tet-o gene expression, has a larger than anticipated toxic profile. Differentially expressed genes in response to DOX were especially enriched in the suppression of cell proliferation processes, including a decrease in cell cycle progression genes (Figures 3, 4). DOX also has been reported to suppress cell proliferation in epithelial-derived cancers (Fife et al., 1998), providing reason to speculate that tetracycline derivatives might be developed as anti-cancer therapeutics (Kroon et al., 1984; van den Bogert et al., 1985, 1986; Mortison et al., 2018). In human epithelial cell lines, DOX reduces cell proliferation, associated with alterations in mitochondrial function (Sourdeval et al., 2006; Chang et al., 2010; Ahler et al., 2013).

Although future studies will be required to unravel the mechanisms by which DOX suppresses cell proliferation, the change in the mIMCD3 transcriptome profile provides important insights. Decreased expression of cytokines and chemokines (Colombo et al., 2018; Koga et al., 2019), together with the attenuation of intracellular signal transduction pathways (cAMP, ERK, and Notch) that are known to regulate cell proliferation (Yamaguchi et al., 2000; Stork and Schmitt, 2002; Kim and Shivdasani, 2011) provide reason to suggest that DOX suppresses the production of autocrine cell growth factors that are required for mIMCD3 proliferation. Additionally, DOX activated the interferon-beta pathway, involving guanylate-binding proteins (GBPs) and interferon activated genes (Lubeseder-Martellato et al., 2002; Klamp et al., 2003; MacMicking, 2004), which is known to inhibit cell proliferation and differentiation (Hertzog et al., 1994; Garrison et al., 1996; Vitale et al., 2006). It will be interesting to learn if either of these pathways can be manipulated to reduce the anti-proliferation effects of DOX. It will be important to corroborate that changes in protein abundance track changes in transcript levels.

The effects of DOX to rapidly induce an anti-proliferative transcriptomic signature in mIMCD3 cells should not be taken as a reason to discontinue use of the otherwise highly effective DOX-based gene induction system. Because it seems likely that all induction agents will have some off-target effects, it more practical to develop strategies to work around the off-target responses. Washing out DOX after gene induction is an obvious remedy. However, future studies will be required to determine the longevity of the DOX response, after withdrawal.

Growth and differentiation of cells *in vitro* can be profoundly influenced by the cell culture medium, nutrients, growth factors, hormones, and other supplements. They were not varied in the present study, and thus should not contribute to differences in the transcriptome profiles between vehicle and DOX treated cells. However, the basal transcriptome profile is likely to be influenced by the supplements in the media. Moreover, potential synergistic actions between DOX and any one of the many supplements is unknowable. Consequently, caution should be exercised in generalizing our results with other studies that use different culture media.

In summary, we have assessed the transcriptomic response to DOX in mIMCD3 at genome-wide resolution. The data base provides an atlas to guide future interpretation of studies using the DOX-inducible gene expression system in renal epithelial cells.

## Data Availability Statement

The datasets presented in this study can be found in online repositories. The names of the repository/repositories and accession number(s) can be found in the article/Supplementary Material.

## Author Contributions

HJ, OW, and PW conceived and designed the studies. HJ, RC, OW, and PW performed the experiments, analyzed the data, and wrote the manuscript. All authors discussed and reviewed the manuscript.

## Conflict of Interest

The authors declare that the research was conducted in the absence of any commercial or financial relationships that could be construed as a potential conflict of interest.

## Publisher’s Note

All claims expressed in this article are solely those of the authors and do not necessarily represent those of their affiliated organizations, or those of the publisher, the editors and the reviewers. Any product that may be evaluated in this article, or claim that may be made by its manufacturer, is not guaranteed or endorsed by the publisher.

## Footnotes

1
http://metascape.org/

2
https://www.ncbi.nlm.nih.gov/geo/query/acc.cgi?&acc=GSE171573

## References

[B1] Aboudehen K. Noureddine L. Cobo-Stark P. Avdulov S. Farahani S. Gearhart M. D. (2017). Hepatocyte nuclear factor-1beta regulates urinary concentration and response to hypertonicity. *J. Am. Soc. Nephrol.* 28 2887–2900. 10.1681/ASN.2016101095 28507058 PMC5619957

[B2] Ahler E. Sullivan W. J. Cass A. Braas D. York A. G. Bensinger S. J. (2013). Doxycycline alters metabolism and proliferation of human cell lines. *PLoS One* 8:e64561. 10.1371/journal.pone.0064561 23741339 PMC3669316

[B3] Amsler K. Ghatani S. Hemmings B. A. (1991). cAMP-dependent protein kinase regulates renal epithelial cell properties. *Am. J. Physiol.* 260(6 Pt 1) C1290–C1299. 10.1152/ajpcell.1991.260.6.C1290 1711777

[B4] Aschauer L. Gruber L. N. Pfaller W. Limonciel A. Athersuch T. J. Cavill R. (2013). Delineation of the key aspects in the regulation of epithelial monolayer formation. *Mol. Cell. Biol.* 33 2535–2550. 10.1128/MCB.01435-12 23608536 PMC3700122

[B5] Barth A. I. M. Kim H. Riedel-Kruse I. H. (2018). Regulation of epithelial migration by epithelial cell adhesion molecule requires its Claudin-7 interaction domain. *PLoS One* 13:e0204957. 10.1371/journal.pone.0204957 30304739 PMC6179577

[B6] Bonnans C. Chou J. Werb Z. (2014). Remodelling the extracellular matrix in development and disease. *Nat. Rev. Mol. Cell Biol.* 15 786–801. 10.1038/nrm3904 25415508 PMC4316204

[B7] Cai Q. Dmitrieva N. I. Ferraris J. D. Brooks H. L. van Balkom B. W. Burg M. (2005). Pax2 expression occurs in renal medullary epithelial cells *in vivo* and in cell culture, is osmoregulated, and promotes osmotic tolerance. *Proc. Natl. Acad. Sci. U.S.A.* 102 503–508. 10.1073/pnas.0408840102 15623552 PMC544323

[B8] Chambers B. E. Gerlach G. F. Clark E. G. Chen K. H. Levesque A. E. Leshchiner I. (2019). Tfap2a is a novel gatekeeper of nephron differentiation during kidney development. *Development* 146:dev172387. 10.1242/dev.172387 31160420 PMC6633607

[B9] Chan S. C. Zhang Y. Shao A. Avdulov S. Herrera J. Aboudehen K. (2018). Mechanism of fibrosis in HNF1B-related autosomal dominant tubulointerstitial kidney disease. *J. Am. Soc. Nephrol.* 29 2493–2509. 10.1681/ASN.2018040437 30097458 PMC6171276

[B10] Chang W. Y. Clements D. Johnson S. R. (2010). Effect of doxycycline on proliferation, MMP production, and adhesion in LAM-related cells. *Am. J. Physiol. Lung Cell. Mol. Physiol.* 299 L393–L400. 10.1152/ajplung.00437.2009 20581100

[B11] Chen Y. Lun A. T. Smyth G. K. (2016). From reads to genes to pathways: differential expression analysis of RNA-Seq experiments using Rsubread and the edgeR quasi-likelihood pipeline. *F1000Researh* 5:1438. 10.12688/f1000research.8987.2 27508061 PMC4934518

[B12] Chukwudi C. U. (2016). rRNA binding sites and the molecular mechanism of action of the tetracyclines. *Antimicrob. Agents Chemother.* 60 4433–4441. 10.1128/AAC.00594-16 27246781 PMC4958212

[B13] Chukwudi C. U. Good L. (2016). Interaction of the tetracyclines with double-stranded RNAs of random base sequence: new perspectives on the target and mechanism of action. *J. Antibiot.* 69 622–630. 10.1038/ja.2015.145 26786504

[B14] Chung E. Deacon P. Park J. S. (2017). Notch is required for the formation of all nephron segments and primes nephron progenitors for differentiation. *Development* 144 4530–4539. 10.1242/dev.156661 29113990 PMC5769624

[B15] Cohen D. M. Chin W. W. Gullans S. R. (1994). Hyperosmotic urea increases transcription and synthesis of Egr-1 in murine inner medullary collecting duct (mIMCD3) cells. *J. Biol. Chem.* 269 25865–25870. 7929290

[B16] Cohen D. M. Gullans S. R. Chin W. W. (1996). Urea signaling in cultured murine inner medullary collecting duct (mIMCD3) cells involves protein kinase C, inositol 1,4,5-trisphosphate (IP3), and a putative receptor tyrosine kinase. *J. Clin. Invest.* 97 1884–1889. 10.1172/JCI118619 8621772 PMC507257

[B17] Colombo M. Mirandola L. Chiriva-Internati M. Basile A. Locati M. Lesma E. (2018). Cancer cells exploit Notch signaling to redefine a supportive cytokine milieu. *Front. Immunol.* 9:1823. 10.3389/fimmu.2018.01823 30154786 PMC6102368

[B18] Commins S. P. Borish L. Steinke J. W. (2010). Immunologic messenger molecules: cytokines, interferons, and chemokines. *J. Allergy Clin. Immunol.* 125(2 Suppl. 2) S53–S72. 10.1016/j.jaci.2009.07.008 19932918

[B19] Coste B. Mathur J. Schmidt M. Earley T. J. Ranade S. Petrus M. J. (2010). Piezo1 and Piezo2 are essential components of distinct mechanically activated cation channels. *Science* 330 55–60. 10.1126/science.1193270 20813920 PMC3062430

[B20] Das A. T. Tenenbaum L. Berkhout B. (2016). Tet-on systems for doxycycline-inducible gene expression. *Curr. Gene Ther.* 16 156–167. 10.2174/1566523216666160524144041 27216914 PMC5070417

[B21] Diaz-Coranguez M. Liu X. Antonetti D. A. (2019). Tight junctions in cell proliferation. *Int. J. Mol. Sci.* 20:5972. 10.3390/ijms20235972 31783547 PMC6928848

[B22] Fife R. S. Sledge G. W. Jr. Roth B. J. Proctor C. (1998). Effects of doxycycline on human prostate cancer cells *in vitro*. *Cancer Lett.* 127 37–41. 10.1016/s0304-3835(98)00003-2 9619856

[B23] Flannery R. J. Kleene N. K. Kleene S. J. (2015). A TRPM4-dependent current in murine renal primary cilia. *Am. J. Physiol. Renal Physiol.* 309 F697–F707. 10.1152/ajprenal.00294.2015 26290373 PMC4609916

[B24] Garrison J. I. Berens M. E. Shapiro J. R. Treasurywala S. Floyd-Smith G. (1996). Interferon-beta inhibits proliferation and progression through S phase of the cell cycle in five glioma cell lines. *J. Neurooncol.* 30 213–223. 10.1007/BF00177272 8943096

[B25] Gomes S. A. Hare J. M. Rangel E. B. (2018). Kidney-derived c-Kit(+) cells possess regenerative potential. *Stem Cells Transl. Med.* 7 317–324. 10.1002/sctm.17-0232 29575816 PMC5866938

[B26] Groenestege W. M. Hoenderop J. G. van den Heuvel L. Knoers N. Bindels R. J. (2006). The epithelial Mg2+ channel transient receptor potential melastatin 6 is regulated by dietary Mg2+ content and estrogens. *J. Am. Soc. Nephrol.* 17 1035–1043. 10.1681/ASN.2005070700 16524949

[B27] Hertzog P. J. Hwang S. Y. Kola I. (1994). Role of interferons in the regulation of cell proliferation, differentiation, and development. *Mol. Reprod. Dev.* 39 226–232. 10.1002/mrd.1080390216 7530016

[B28] Hilgendorf K. I. Johnson C. T. Jackson P. K. (2016). The primary cilium as a cellular receiver: organizing ciliary GPCR signaling. *Curr. Opin. Cell Biol.* 39 84–92. 10.1016/j.ceb.2016.02.008 26926036 PMC4828300

[B29] Ichikawa-Tomikawa N. Sugimoto K. Satohisa S. Nishiura K. Chiba H. (2011). Possible involvement of tight junctions, extracellular matrix and nuclear receptors in epithelial differentiation. *J. Biomed. Biotechnol.* 2011:253048. 10.1155/2011/253048 22162632 PMC3227411

[B30] Jedroszka D. Orzechowska M. Hamouz R. Gorniak K. Bednarek A. K. (2017). Markers of epithelial-to-mesenchymal transition reflect tumor biology according to patient age and Gleason score in prostate cancer. *PLoS One* 12:e0188842. 10.1371/journal.pone.0188842 29206234 PMC5714348

[B31] Kang K. Huang L. Li Q. Liao X. Dang Q. Yang Y. (2019). An improved Tet-on system in microRNA overexpression and CRISPR/Cas9-mediated gene editing. *J. Anim. Sci. Biotechnol.* 10:43. 10.1186/s40104-019-0354-5 31198556 PMC6556963

[B32] Kim T. H. Shivdasani R. A. (2011). Notch signaling in stomach epithelial stem cell homeostasis. *J. Exp. Med.* 208 677–688. 10.1084/jem.20101737 21402740 PMC3137787

[B33] Kistner A. Gossen M. Zimmermann F. Jerecic J. Ullmer C. Lubbert H. (1996). Doxycycline-mediated quantitative and tissue-specific control of gene expression in transgenic mice. *Proc. Natl. Acad. Sci. U.S.A.* 93 10933–10938. 10.1073/pnas.93.20.10933 8855286 PMC38261

[B34] Klamp T. Boehm U. Schenk D. Pfeffer K. Howard J. C. (2003). A giant GTPase, very large inducible GTPase-1, is inducible by IFNs. *J. Immunol.* 171 1255–1265. 10.4049/jimmunol.171.3.1255 12874213

[B35] Koga Y. Tsurumaki H. Aoki-Saito H. Sato M. Yatomi M. Takehara K. (2019). Roles of cyclic AMP response element binding activation in the ERK1/2 and p38 MAPK signalling pathway in central nervous system, cardiovascular system, osteoclast differentiation and mucin and cytokine production. *Int. J. Mol. Sci.* 20:1346. 10.3390/ijms20061346 30884895 PMC6470985

[B36] Kroon A. M. Dontje B. H. Holtrop M. Van den Bogert C. (1984). The mitochondrial genetic system as a target for chemotherapy: tetracyclines as cytostatics. *Cancer Lett.* 25 33–40. 10.1016/s0304-3835(84)80023-3 6518450

[B37] Kume T. Deng K. Hogan B. L. (2000). Murine forkhead/winged helix genes Foxc1 (Mf1) and Foxc2 (Mfh1) are required for the early organogenesis of the kidney and urinary tract. *Development* 127 1387–1395. 10704385 10.1242/dev.127.7.1387

[B38] Larrayoz I. M. de Luis A. Rua O. Velilla S. Cabello J. Martinez A. (2012). Molecular effects of doxycycline treatment on pterygium as revealed by massive transcriptome sequencing. *PLoS One* 7:e39359. 10.1371/journal.pone.0039359 22724003 PMC3378547

[B39] Lashhab R. Rumley A. C. Arutyunov D. Rizvi M. You C. Dimke H. (2019). The kidney anion exchanger 1 affects tight junction properties via claudin-4. *Sci. Rep.* 9:3099. 10.1038/s41598-019-39430-9 30816203 PMC6395713

[B40] LeBleu V. S. Taduri G. O’Connell J. Teng Y. Cooke V. G. Woda C. (2013). Origin and function of myofibroblasts in kidney fibrosis. *Nat. Med.* 19 1047–1053. 10.1038/nm.3218 23817022 PMC4067127

[B41] Leiz J. Schmidt-Ott K. M. (2019). Claudins in the renal collecting duct. *Int. J. Mol. Sci.* 21:221. 10.3390/ijms21010221 31905642 PMC6981911

[B42] Liu X. Vien T. Duan J. Sheu S. H. DeCaen P. G. Clapham D. E. (2018). Polycystin-2 is an essential ion channel subunit in the primary cilium of the renal collecting duct epithelium. *Elife* 7:e33183. 10.7554/eLife.33183 29443690 PMC5812715

[B43] Lu H. Jiang W. Yang H. Qin Z. Guo S. E. Hu M. (2017). Doxycycline affects gene expression profiles in aortic tissues in a rat model of vascular calcification. *Microvasc. Res.* 114 12–18. 10.1016/j.mvr.2017.04.007 28546078

[B44] Lubeseder-Martellato C. Guenzi E. Jorg A. Topolt K. Naschberger E. Kremmer E. (2002). Guanylate-binding protein-1 expression is selectively induced by inflammatory cytokines and is an activation marker of endothelial cells during inflammatory diseases. *Am. J. Pathol.* 161 1749–1759. 10.1016/S0002-9440(10)64452-5 12414522 PMC1850787

[B45] MacMicking J. D. (2004). IFN-inducible GTPases and immunity to intracellular pathogens. *Trends Immunol.* 25 601–609. 10.1016/j.it.2004.08.010 15489189

[B46] Matter K. Aijaz S. Tsapara A. Balda M. S. (2005). Mammalian tight junctions in the regulation of epithelial differentiation and proliferation. *Curr. Opin. Cell Biol.* 17 453–458. 10.1016/j.ceb.2005.08.003 16098725

[B47] Mortison J. D. Schenone M. Myers J. A. Zhang Z. Chen L. Ciarlo C. (2018). Tetracyclines modify translation by targeting key human rRNA substructures. *Cell Chem. Biol.* 25 1506–1518.e13. 10.1016/j.chembiol.2018.09.010 30318461 PMC6309532

[B48] Mykytyn K. Askwith C. (2017). G-protein-coupled receptor signaling in cilia. *Cold Spring Harb. Perspect. Biol.* 9:a028183. 10.1101/cshperspect.a028183 28159877 PMC5585845

[B49] Patro R. Duggal G. Love M. I. Irizarry R. A. Kingsford C. (2017). Salmon provides fast and bias-aware quantification of transcript expression. *Nat. Methods* 14 417–419. 10.1038/nmeth.4197 28263959 PMC5600148

[B50] Ransick A. Lindstrom N. O. Liu J. Zhu Q. Guo J. J. Alvarado G. F. (2019). Single-cell profiling reveals sex, lineage, and regional diversity in the mouse kidney. *Dev. Cell* 51 399–413.e7. 10.1016/j.devcel.2019.10.005 31689386 PMC6948019

[B51] Rauchman M. I. Nigam S. K. Delpire E. Gullans S. R. (1993). An osmotically tolerant inner medullary collecting duct cell line from an SV40 transgenic mouse. *Am. J. Physiol.* 265(3 Pt 2) F416–F424. 10.1152/ajprenal.1993.265.3.F416 8214101

[B52] Reboredo M. Kramer M. G. Smerdou C. Prieto J. De Las Rivas J. (2008). Transcriptomic effects of Tet-on and mifepristone-inducible systems in mouse liver. *Hum. Gene Ther.* 19 1233–1247. 10.1089/hum.2008.057 19025414

[B53] Ribes D. Fischer E. Calmont A. Rossert J. (2003). Transcriptional control of epithelial differentiation during kidney development. *J. Am. Soc. Nephrol.* 14(Suppl. 1) S9–S15. 10.1097/01.asn.0000067647.05964.9f 12761232

[B54] Robinson M. D. McCarthy D. J. Smyth G. K. (2010). edgeR: a bioconductor package for differential expression analysis of digital gene expression data. *Bioinformatics* 26 139–140. 10.1093/bioinformatics/btp616 19910308 PMC2796818

[B55] Schlimpert M. Lagies S. Budnyk V. Muller B. Walz G. Kammerer B. (2018). Metabolic phenotyping of Anks3 depletion in mIMCD-3 cells – a putative nephronophthisis candidate. *Sci. Rep.* 8:9022. 10.1038/s41598-018-27389-y 29899363 PMC5998149

[B56] Schwab K. Patterson L. T. Aronow B. J. Luckas R. Liang H. C. Potter S. S. (2003). A catalogue of gene expression in the developing kidney. *Kidney Int.* 64 1588–1604. 10.1046/j.1523-1755.2003.00276.x 14531791

[B57] Siroky B. J. Kleene N. K. Kleene S. J. Varnell C. D. Jr. Comer R. G. Liu J. (2017). Primary cilia regulate the osmotic stress response of renal epithelial cells through TRPM3. *Am. J. Physiol. Renal Physiol.* 312 F791–F805. 10.1152/ajprenal.00465.2015 28122715 PMC5407065

[B58] Slaats G. G. Wheway G. Foletto V. Szymanska K. van Balkom B. W. Logister I. (2015). Screen-based identification and validation of four new ion channels as regulators of renal ciliogenesis. *J. Cell Sci.* 128 4550–4559. 10.1242/jcs.176065 26546361 PMC4696498

[B59] Sourdeval M. Lemaire C. Brenner C. Boisvieux-Ulrich E. Marano F. (2006). Mechanisms of doxycycline-induced cytotoxicity on human bronchial epithelial cells. *Front. Biosci.* 11:3036–3048. 10.2741/2031 16720374

[B60] Stadnyk A. W. (1994). Cytokine production by epithelial cells. *FASEB J.* 8 1041–1047. 10.1096/fasebj.8.13.7926369 7926369

[B61] Stork P. J. Schmitt J. M. (2002). Crosstalk between cAMP and MAP kinase signaling in the regulation of cell proliferation. *Trends Cell Biol.* 12 258–266. 10.1016/s0962-8924(02)02294-8 12074885

[B62] Strait K. A. Stricklett P. K. Chapman M. Kohan D. E. (2010). Characterization of vasopressin-responsive collecting duct adenylyl cyclases in the mouse. *Am. J. Physiol. Renal Physiol.* 298 F859–F867. 10.1152/ajprenal.00109.2009 19955190 PMC2853316

[B63] Sukhatme V. P. Cao X. M. Chang L. C. Tsai-Morris C. H. Stamenkovich D. Ferreira P. C. (1988). A zinc finger-encoding gene coregulated with c-fos during growth and differentiation, and after cellular depolarization. *Cell* 53 37–43. 10.1016/0092-8674(88)90485-0 3127059

[B64] Torban E. Eccles M. R. Favor J. Goodyer P. R. (2000). PAX2 suppresses apoptosis in renal collecting duct cells. *Am. J. Pathol.* 157 833–842. 10.1016/S0002-9440(10)64597-X 10980123 PMC1885702

[B65] Valkova N. Kultz D. (2006). Constitutive and inducible stress proteins dominate the proteome of the murine inner medullary collecting duct-3 (mIMCD3) cell line. *Biochim. Biophys. Acta* 1764 1007–1020. 10.1016/j.bbapap.2006.03.007 16713411

[B66] van den Bogert C. Dontje B. H. Holtrop M. Melis T. E. Romijn J. C. van Dongen J. W. (1986). Arrest of the proliferation of renal and prostate carcinomas of human origin by inhibition of mitochondrial protein synthesis. *Cancer Res.* 46 3283–3289. 3011245

[B67] van den Bogert C. Dontje B. H. Kroon A. M. (1985). The antitumour effect of doxycycline on a T-cell leukaemia in the rat. *Leuk. Res.* 9 617–623. 10.1016/0145-2126(85)90142-0 3874329

[B68] Vitale G. de Herder W. W. van Koetsveld P. M. Waaijers M. Schoordijk W. Croze E. (2006). IFN-beta is a highly potent inhibitor of gastroenteropancreatic neuroendocrine tumor cell growth *in vitro*. *Cancer Res.* 66 554–562. 10.1158/0008-5472.CAN-05-3043 16397272

[B69] Wei Q. Khan I. K. Ding Z. Yerneni S. Kihara D. (2017). NaviGO: interactive tool for visualization and functional similarity and coherence analysis with gene ontology. *BMC Bioinformatics* 18:177. 10.1186/s12859-017-1600-5 28320317 PMC5359872

[B70] Wishart J. A. Hayes A. Wardleworth L. Zhang N. Oliver S. G. (2005). Doxycycline, the drug used to control the tet-regulatable promoter system, has no effect on global gene expression in *Saccharomyces cerevisiae*. *Yeast* 22 565–569. 10.1002/yea.1225 15942933

[B71] Yamaguchi T. Pelling J. C. Ramaswamy N. T. Eppler J. W. Wallace D. P. Nagao S. (2000). cAMP stimulates the *in vitro* proliferation of renal cyst epithelial cells by activating the extracellular signal-regulated kinase pathway. *Kidney Int.* 57 1460–1471. 10.1046/j.1523-1755.2000.00991.x 10760082

[B72] Yu Z. Kong Q. Kone B. C. (2013). Aldosterone reprograms promoter methylation to regulate alphaENaC transcription in the collecting duct. *Am. J. Physiol. Renal Physiol.* 305 F1006–F1013. 10.1152/ajprenal.00407.2013 23926181 PMC3798741

[B73] Zhao B. Ye X. Yu J. Li L. Li W. Li S. (2008). TEAD mediates YAP-dependent gene induction and growth control. *Genes Dev.* 22 1962–1971. 10.1101/gad.1664408 18579750 PMC2492741

[B74] Zhou Y. Zhou B. Pache L. Chang M. Khodabakhshi A. H. Tanaseichuk O. (2019). Metascape provides a biologist-oriented resource for the analysis of systems-level datasets. *Nat. Commun.* 10:1523. 10.1038/s41467-019-09234-6 30944313 PMC6447622

